# Economic Impact of Dengue Virus in Mexico Over One Decade: A Systematic Literature Review (2010–2020)

**DOI:** 10.1002/hpm.70096

**Published:** 2026-07-28

**Authors:** Gilberto Sánchez‐González, Luz del C. Hernández‐Hernández, Elaine Gallagher

**Affiliations:** ^1^ Centro de Investigación Sobre Enfermedades Infecciosas Instituto Nacional de Salud Pública Cuernavaca Morelos México; ^2^ Independent Consultant Cuernavaca Morelos México; ^3^ Takeda Pharmaceuticals International AG Zurich Switzerland

**Keywords:** burden, dengue, economic, Mexico, review

## Abstract

**Background:**

Dengue is a major mosquito‐borne viral disease worldwide, imposing significant healthcare burden on governments in countries where it is endemic, such as Mexico. This systematic review focused on studies reporting the cost of dengue in Mexico during 2010–2020. To the best of our knowledge, this is the first review discussing this topic in Mexico. We analysed all published direct costs associated with care, prevention, and surveillance, as well as indirect costs.

**Methods:**

A comprehensive online search was conducted using Embase, MEDLINE, Evidence‐Based Medicine Reviews, SciELO, and LILACS. Articles reporting cost data published during 2010–2020 in English and Spanish were included. Reports from the National Ministry of Health and grey literature during the same period were also reviewed. Following the PICOS (Population, Intervention, Comparison, Outcome and Study design) framework, articles reporting direct and indirect costs were included. The National Health Service Wales tool was used to assess the quality of the studies included.

**Results:**

Seven articles fulfiled the inclusion criteria. All articles reported the nationwide cost of dengue, and three focused on specific Mexican states. The dengue‐related economic burden was substantial, peaking in 2013, with annual costs of $176.82 million (adjusted present value, $281.60 million). All the searches were verified in January 2023.

**Conclusions:**

The economic burden of dengue in Mexico is high and is further impacted by outbreaks, resulting in significant costs to the healthcare system and productivity losses. The heterogeneity of the methodological approaches in the cost studies reviewed makes it a challenge to compare them.

## Highlights

- We present SLR results on the economic burden of dengue in Mexico.
- The SLR covers the years 2010–2020.
- Seven articles were considered; all seven reported the cost of illness nationwide.
- The dengue‐related burden in 2013 was $281.60 million.

## 1 Introduction

Dengue is a viral disease caused by a single‐stranded flavivirus transmitted to humans through the bite of infected female mosquito vectors (i.e., *Aedes aegypti* and, probably, *Aedes albopictus*) [1, 2]. A 30‐fold increase in the incidence of dengue was observed between 1960 and 2010, making it the fastest spreading mosquito‐borne viral disease worldwide [3, 4]. In addition to the increase in incidence, the geographical areas where dengue is transmitted have expanded in recent years, mainly because of population growth, unplanned urbanisation, inefficient mosquito control, frequent international travel (wherein travellers carry the virus from endemic to nonendemic regions), and global warming [5].

Dengue affects more than 100 million people a year worldwide. The Americas reported three million cases in 2019, of which 8% were in Mexico [6]. Dengue is a reportable disease in Mexico [7] and endemic in specific regions, with most cases reported in the south of the country, where all four serotypes are present [7, 8]. Despite the known impact of dengue, there is substantial uncertainty about the magnitude of the disease burden and cost, particularly with respect to the number of symptomatic infections. Furthermore, despite efforts towards disease prevention and vector control over the past two decades, the number of cases has increased from 21,615 in 2000 to 264,898 in 2019 [9]. A major outbreak was reported over the last 2 years in Mexico, with 56,333 confirmed cases in 2023; of these, 29,810 were severe. The second outbreak, which was recorded in 2024, comprised 125,160 confirmed cases and 68,240 severe cases. Circulation of all four serotypes was reported in both outbreaks. Mortality per 100 cases (studied and validated by an expert committee) was 1.85 (i.e., 491 fatal cases) in 2023 and 0.84 (i.e., 478 fatal cases) in 2024. According to the Ministry of Health (MoH), the disease incurs significant economic burden for both public and private hospitals [10].

### 1.1 Symptoms and Clinical Presentation

Although the present study has its initial starting year as 2010, the possibility that some studies from 2010 were still based on the 1990 dengue classification was not ruled out, although it is important to note that classifications according to 1997 [11] and 2009 [4] may occur. The 1997 WHO classification for dengue is as follows: grade I, fever with nonspecific symptoms, the only haemorrhagic sign being a positive tourniquet test or easy bruising; grade II, spontaneous bleeding plus grade I manifestations, usually skin or other hemorrhages; grade III, circulatory failure with a rapid, weak pulse, narrow pulse pressure, or hypotension, cold skin, and restlessness; grade IV, severe shock with undetectable pulse or blood pressure [11]. Owing to changes in the epidemiology of dengue over time, use of the existing WHO classification may lead to conflicting results. In the 2009 edition of the guidelines, symptomatic dengue virus infections were grouped into three categories: undifferentiated fever, dengue fever, and dengue haemorrhagic fever. Of these, dengue haemorrhagic fever was further classified into four severity grades, with grades III and IV being defined as (integrated into) dengue shock syndrome. In this review, we maintained the definitions and outcomes established in the 1997 guidelines, as presented in the included studies.

The MoH has published laboratory protocols for serological confirmation of dengue and disseminates weekly surveillance data. Furthermore, in collaboration with the 32 state health services and other health organisations (Mexican Institute of Social Security [IMSS] and Institute of Social Security and Services for State Workers [ISSSTE]), the MoH has developed guidelines for vector control and dengue surveillance systems [12]. Probable and confirmed dengue cases must be reported weekly, whereas cases of dengue haemorrhagic fever and dengue‐related deaths must be reported within 24 h. A web‐based epidemiological surveillance platform, implemented in 2008, enables healthcare workers to enter data directly into the national database, preventing underestimation. In 2021, this platform was modified, coinciding with the onset of the COVID‐19 pandemic, wherein data input was modified to account for cases without a confirmed clinical diagnosis. In addition, other means of estimating positive cases were also employed.

The 2010–2020 pre‐pandemic decade was selected to provide a robust baseline for examining the economic burden of dengue in Mexico. This period enables meaningful comparisons with post‐pandemic trends and allows assessment of health system capacity prior to the introduction of new preventive tools, including vaccines. Most dengue‐endemic countries, including Mexico, are currently affected by budgetary constraints due to evolving economic dynamics, climate change, and shifting health priorities. Documenting both direct and indirect costs supports the case for continued investment in early surveillance, vector control, and timely outbreak responses, thereby guiding effective resource allocation, therefore, for the present study, we considered the decade with a cut‐off in 2020 [13].

The high incidence of dengue cases has an impact not only from an epidemiological perspective, but also from an economic perspective, in terms of the treatment cost [14, 15]. In Mexico, the overall economic impact of dengue from 2012 to 2016 was estimated at US$699 million [16]. Additionally, the disease is often underrecognized or misdiagnosed owing to its broad spectrum of clinical presentations. Therefore, the actual cost of dengue disease may be largely underestimated [17]. However, the significant quantity of cumulative evidence enables us to perform a detailed analysis. The objective of the present study was to conduct a systematic literature review (SLR) of published studies on the costs associated with dengue in Mexico. As shown in the Supporting Information S1: (S1 Table 1 to Table 5), the search strategy considers the direct medical costs of dengue fever, haemorrhagic fever, dengue with alarm signs, or severe dengue. The study categorises the healthcare costs into inpatient (hospitalisation, emergency room, and intensive care unit [ICU]), outpatient (ambulatory), and preventive care (vector control and outbreak control).

## 2 Methods

### 2.1 Research Question

Understanding the cost of dengue is crucial as it helps policymakers to effectively allocate resources, plan public health interventions, and assess the economic burden of the disease on healthcare systems and society. Accurate cost data also support cost‐effectiveness analysis for prevention and control strategies. This SLR aimed to determine the cost of dengue in Mexico during the pre‐pandemic decade of 2010–2020.

### 2.2 Literature Search and Study Identification

This SLR was conducted according to the Cochrane Handbook for Systematic Reviews of Interventions, and the results were reported according to the Preferred Reporting Items for Systematic Reviews and Meta‐Analysis (PRISMA) guidelines.

To answer the research question, all available published direct costs (of care, prevention, and surveillance) were analysed. Indirect costs were also assessed. The databases, including Embase, MEDLINE, Evidence‐Based Medicine Reviews, SciELO, and LILACS, were searched via the OVID platform on March 17, 2020, using a predefined search strategy to identify studies on the cost of dengue in Mexico. The search strategy included a combination of free‐text terms and subject headings, including Medical Subject Headings (MeSH) terms, to identify relevant articles. The results of the search strategy are shown in the Supporting Information S1: (S1 Table 1–5).

### 2.3 Data Extraction and Risk of Bias

A structured data extraction form was developed for the SLR in Microsoft Excel. Key data from each eligible study were extracted by recording data from the original reports onto the data extraction form. Each of the selected articles was fully assessed to determine the quality of the eligible studies using a recognized quality assessment tool [18]. This ensured the inclusion of high‐quality data to draw reliable conclusions that would inform decision‐making.

To augment the findings of the database search and reduce publication bias, additional data were captured from the grey literature, which encompassed international, national, and regional databases such as the WHO Library Information System (WHOLIS), the Pan‐American Health Organisation (PAHO), the International Society for Pharmacoeconomics and Outcomes and Research (ISPOR), the Instituto Nacional de Salud Pública (INSP), and the Virtual Health Library (VHL).

### 2.4 Inclusion and Exclusion Criteria

A total of 414 records were identified from the database searches. After applying the filters and deduplication, 412 records were included for title and abstract screening. Each of the identified abstracts was reviewed independently by two analysts. The PICOS (Population, Intervention, Comparison, Outcomes, and Study design) framework was applied, and abstracts were further assessed with full‐text screening if deemed eligible for inclusion based on the criteria detailed in Table 1.

**TABLE 1:** Inclusion and exclusion criteria used in the systematic literature review.

	Inclusion criteria	Exclusion criteria
Population	Individuals of all ages with suspected or confirmed dengue reported in Mexico	Animal and vector data
Intervention	SLR not restricted by intervention criteria	NA
Comparison	SLR not restricted by comparison criteria	
Outcomes	Cost of illness and economic burden of dengue and/or severe dengue to patients and health services (according to payer type), and/or societal perspective, such as – Direct medical costs – Direct nonmedical costs – Indirect societal costs Costs for outbreak control	Studies published outside the date limits: – Cost data: between 2010 and 2020
Study design	Cost‐of‐illness studies Real‐world studies, such as database analyses and longitudinal observational studies SLRs if a meta‐analysis is included	– Publications that do not clearly outline the methods and sources for data collection/analysis – Economic evaluation studies – In vitro studies – Clinical trials that do not report baseline results – News and opinion articles

Abbreviations: NA: not available; SLR: systematic literature review.

The adjusted present value (APV) of the cost was normalised using the inflation calculator developed by the National Institute of Statistics and Geography (INEGI), based on the historical national consumer price index [19].

### 2.5 Management of Uncertainty

To address uncertainty, all reported direct medical and non‐medical costs, as well as indirect costs, were inflation‐adjusted to 2020 values (Table S6). Conversion was standardized using an exchange rate of 20 Mexican pesos per United States dollar (USD), consistent with the consumer price index methodology.

## 3 Results

**FIGURE 1:**
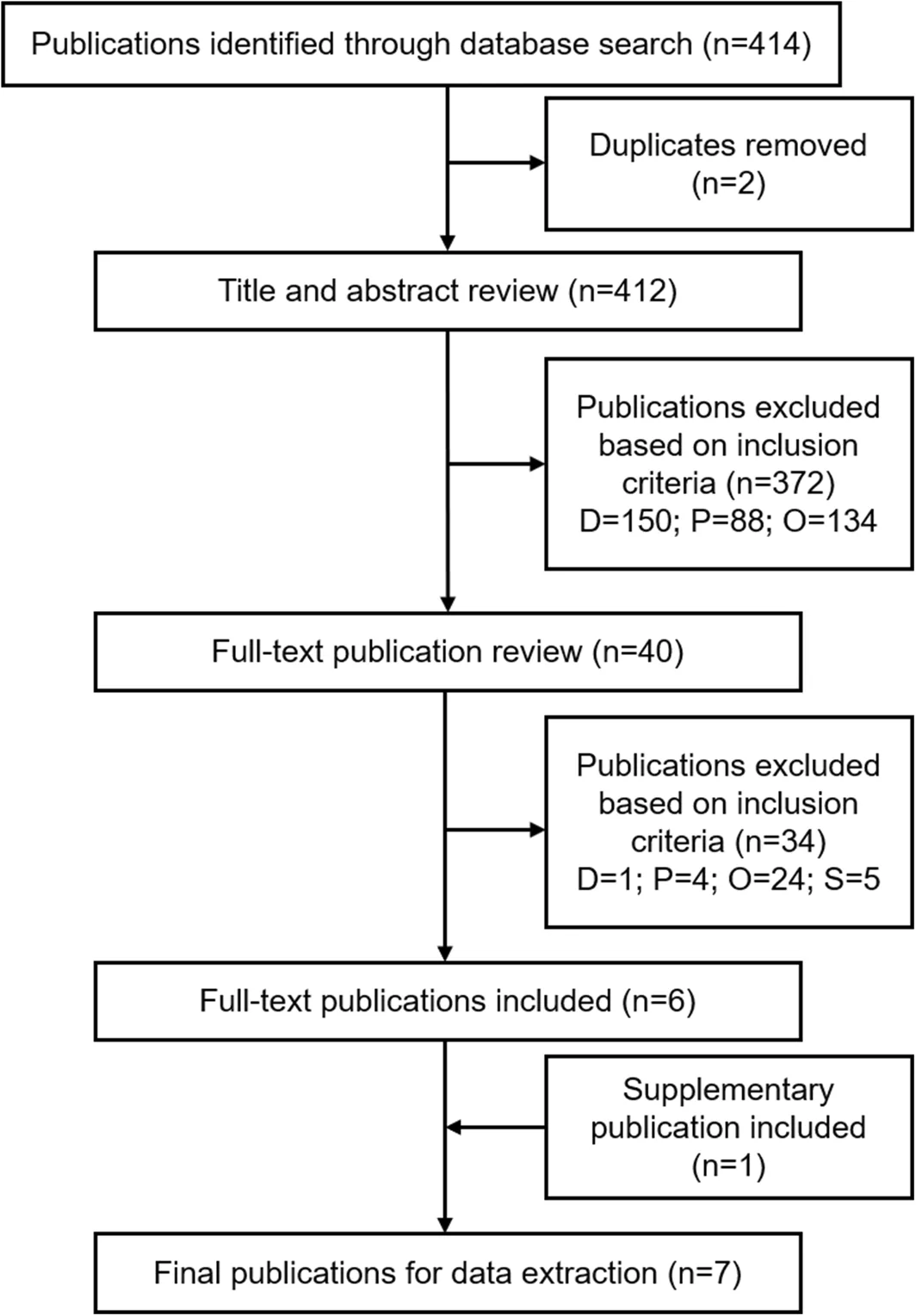
PRISMA Study Flowchart. The chain search produced 414 hits, from which two duplicates were removed and 372 were excluded based on the inclusion criteria. From the remaining 40 full‐text publications, 34 were removed based on the inclusion criteria, resulting in six eligible full‐text publications, and one additional supplementary publication. D: duplicate; O: outcome; P: population; S: study design.

Forty full‐text publications were reviewed according to the PICOS criteria, and of these, six were selected for inclusion in the SLR. A supplementary publication from the American Journal of Tropical Medicine and Hygiene was also included, but no further studies from the grey literature were included. Therefore, a total of seven articles were included for data extraction. The results of the literature search are presented as a PRISMA flowchart in Figure 1.

Seven cost‐of‐illness studies were included to investigate the economic burden of dengue in Mexico. Most studies presented different cost categories (direct medical and nonmedical costs and indirect costs), whereas Tiga et al. [20] grouped costs into total annual costs, including direct and indirect costs. A summary of the estimated average economic burden of dengue per episode, as reported in the selected studies, is presented in Table 2. This summary was constructed using only the available information extracted from the individual data presented in Table S8.

**TABLE 2:** Summary of estimated average cost burden of dengue per episode or per year (mean [range])^a^.

Subgroup	Cost type	Direct medical cost (USD)	Direct nonmedical cost (USD)	Indirect cost (USD)	Total direct cost (million per year) (USD)	Total indirect cost (million per year) (USD)
Outpatient	Per case	103.96 [17.59–309.52]	15.24 [ND]	245.04 [ND]	12.22 [4.76–38.53]	—
	Primary‐level hospital (per day)	93.33 [ND]	—	—	—	—
	Secondary‐level hospital (per day)	106.34 [ND]	—	—	—	—
	Tertiary‐level hospital (per day)	110.32 [ND]	—	—	—	—
	Per case acute dengue	—	—	—	45.92 [ND]	17.38 [14.15–20.60]
Inpatient	Per case	620.35 [103.29–1776.14]	151.86 [93.05–193.70]	304.23 [102.97–660.08]	34.70 [10.67–71.71]	—
	Primary‐level hospital (per day)	256.70 [ND]	—	—	—	—
	Secondary‐level hospital (per day)	267.82 [ND]	—	—	—	—
	Tertiary‐level hospital (per day)	346.31 [ND]	—	—	—	—
	Per case acute dengue	—	—	—	25.12 [ND]	3.02 [ND]
Intensive care unit	Per case	7957 [5790–10,124]	—	240.17 [165.75–314.59]	6.48 [2.15–10.81]	—

Abbreviations: ND: No Data; USD: United States Dollar.

^a^ These costs are from S1 Tables 8‐9, considering real (nonestimated) costs.

A summary of estimated costs, as detailed in Table 2, is as follows: For outpatients, the mean direct medical cost per case was $103.96 (range $17.59–$309.52), with nonmedical costs of $15.24 and indirect costs of $245.04. The total annual direct cost for outpatients was $12.22 million (range $4.76–$38.53 million). For inpatients, the mean direct medical cost was $620.35 (range $103.29–$1776.14), nonmedical cost $151.86 (range $93.05–$193.70), and indirect cost $304.23 (range $102.97–$660.08), resulting in a total annual direct cost of $34.70 million (range $10.67–$71.71 million). Despite limitations in the availability of data, costs related to ICU admissions represented the highest burden. See S1 Table 8–9 for detailed study characteristics.

Shepard et al. [21] reported total annual costs for inpatient, outpatient, and fatal cases, as well as surveillance and control vector costs in 2010 and 2011. Total outpatient costs (reported as cost [APV]) were higher in 2010 ($123.46 [$211.2] million) than in 2011 ($45.61 [$78.0] million). Costs were higher in 2010 than in 2011 ($274.7 [$470.0] vs. $206.56 [$353.4] million, aggregate costs). In 2010, direct medical costs were associated with the highest burden ($89.64 [$153.37] million) compared with indirect costs in 2011 ($98.55 [$168.61] million). The authors adopted a societal perspective and reported direct medical and non‐medical costs, as well as indirect costs, for both outpatient and inpatient care. Total costs per dengue case were lower in outpatients than in inpatients ($569.80 [$974.90] vs. $1417.47 [$2425.29]). The highest expense per outpatient dengue case was direct medical costs ($309.52 [$529.58]), while costs of an inpatient case were mainly driven by higher indirect costs ($660.08 [$1129.39]).

Tiga et al. [20] reported direct medical, indirect, and total costs for acute dengue cases (outpatients and inpatients) and indirect costs for fatal acute dengue cases. Outpatients had higher annual direct medical and indirect costs (reported as cost [APV]) compared with inpatients with acute dengue ($45.92 [$66.18] million vs. $25.12 [$36.20] million and $14.15 [$20.39] million vs. $3.02 [$4.35] million, respectively). Fatal acute dengue costs were driven mainly by indirect costs ($8.43 [$12.15] million per year). Acute dengue was associated with high costs (a total of $96.64 [$139.28]; $74.97 [$108.05] − $232.26 [$334.75] million per year).

In 2012, Undurraga et al. [22] reported direct medical and nonmedical, indirect, and total costs for outpatients and inpatients from a societal perspective. Direct medical costs increased with the hospital level. For both outpatient and inpatients, costs (reported as cost [APV]) were higher in tertiary‐level hospitals ($110.82 [$163.90] per outpatient visit and $346.31 [$512.19] per bed day, respectively), followed by secondary‐level hospitals ($106.34 [$157.27] per outpatient visit and $267.82 [$396.10] per bed day, respectively) and primary‐level hospitals ($93.33 [$138.03] per outpatient visit and $256.70 [$379.65] per bed day, respectively). Age did not appear to influence costs, as bed estimates per outpatient visit and per bed day were similar between patients aged 5–14 years and those aged 15–18 years ($72.97 [$107.92] per outpatient visit and $267.29 [$395.32 APV] per bed day, respectively).

For inpatients, the total cost was $1477.00 ($2184.48) per episode, the direct medical cost was $1124.65 ($1663.35), the direct nonmedical cost was $193.70 ($286.48), and the indirect cost was $159.23 ($235.50). For outpatients, the total cost was $502.00 ($742.45) per episode, the direct medical cost was $281.72 ($416.66), the direct nonmedical cost was $102.44 ($151.50), and the indirect cost was $118.03 ($174.56). Both fatal and nonfatal dengue were associated with a high burden [22]. Outpatient episodes in nonfatal dengue incurred an annual cost of $165.9 ($245.36) million ($151.26 [$223.71] million to $256.78 [$379.77] million) in 2011, while inpatient episodes in nonfatal dengue amounted to $212.10 ($313.69) million ($183.18 [$270.92] million to $397.48 [$587.87] million) in 2010. The average cost of fatal dengue (both outpatient and inpatient episodes included) was $8.43 ($12.46) million ($5.29 [$7.82] million to $12.10 [$17.89] million) during 2010–2011 [22].

Zubieta‐Zavala et al. reported annual costs from 2012 to 2016 in 16 Mexican states from the perspectives of the healthcare system and patients [16]. The highest costs were generated when hospitalization was necessary. In 2012, the average total cost of dengue (APV) was $5,492,287 ($7,161,942) ($2,697,850 [$3,517,996] to $10,148,163 [$13,233,204), of which $5,121,860 ($6,678,905; $2,482,217 [$3,236,] to $9,550,134 [$12,453,374]) was reported for hospitalized cases. A similar result was observed from the healthcare perspective. In 2012, the average total cost of dengue was $49,092,680 ($64,016,854), of which $32,569,467 ($42,470,584) was for inpatients. This study reported costs with and without expansion factors. For example, in 2012, costs without applying an expansion factor were estimated at $160,861,018 ($209,762,767), which was lower than the estimated value with a factor applied ($198,491,321 [$258,832,682] when health facilities were concerned and $240,170,653 [$313,182,531] when all symptoms were included). This is a strong indicator that the burden of dengue is most likely underestimated in Mexico.

In another work by Zubieta‐Zavala et al., ideal and real costs were reported from both the Secretariat of Health and the IMSS perspectives, with ICU patients experiencing the highest costs (for both ideal and real costs) [23]. The Secretariat of Health reported the highest average real costs for ICU patients ($5790.00 [$8345.12] per case), followed by inpatients ($530.16 [$764.11] per case) and outpatients ($35.20 [$50.73] per case). Similarly, the IMSS reported high real costs for ICU patients, with direct medical costs in 2014 averaging $10,123.79 ($14,591.37) per case. However, these costs were almost twice those reported by the Secretariat of Health. In both systems, patients and caregivers combined reported large indirect real costs (reported as cost [APV]) ($314.59 [$453.41] per case from the IMSS perspective and $165.75 [$238.89] per case from the Secretariat of Health perspective).

Legoretta‐Soberanis et al. [24] did not report the year of costing; therefore, the normalisation for 2020 values could not be carried out. Their study included patients from Acapulco, Costa Grande, and Costa Chica, where the average cost of hospitalised patients in all treatment locations was $514.00 ($707.46) per case. Only one publication [21] reported outbreak costs. The authors reported the total annual economic cost for surveillance and vector control from a societal perspective in 2010 and 2011, amounting to $86.07 ($137.07) million and $98.55 ($156.95) million, respectively. Outbreak costs accounted for a large proportion of the total aggregate economic costs reported. In 2010, more than 30% of the total cost (outbreak costs: $86.07 [$137.07] million out of a total cost of $274.70 [$437.48] million) was attributed to outbreak costs, which increased in 2011, to nearly 48% of the total cost (outbreak cost: $98.55 [$156.95] million of a total cost of $206.56 [$328.96] million).

As mentioned previously, Zubieta‐Zavala et al. conducted two retrospective studies. In the study published in 2016 [16], the number of hospital days reported by patients across all settings (outpatient, inpatient, and ICU) amounted to 7 days per dengue case. In the second study, published in 2018 [23], the total number of sick days was 11.5 days for patients with dengue fever and 14.2 days for patients with dengue haemorrhagic fever across all settings considered (outpatient, inpatient, and ICU).

Legorreta‐Soberanis et al. [24] reported the highest number of days lost due to illness using data collected from 2009 to 2013 by the ISSSTE. Inpatients from Acapulco, Costa Grande, and Costa Chica collectively lost an average of 17.3 days (± 9.3 days) due to illness. The burden of dengue was highest in Costa Chica, where patients lost an average of 11.6 days for inpatients and outpatients.

Shepard et al. [21] estimated the annual cost of dengue to be $274.7 ($437.48) million in $2010 and $206.56 ($328.96) million in 2011. The costs ranged from $160.86 ($264.53) million in $2012 to $138.70 ($199.90) million in 2016, peaking at $176.82 ($281.60) million in 2013 [16]. Although a decreasing trend in total annual costs was observed, the burden of dengue remained high in Mexico. Furthermore, as incidence rates have recently increased, the economic impact is likely to be substantial; therefore, studies investigating this impact in recent years are warranted. The burden of dengue was also significantly impacted by outbreaks, costing up to $98.55 ($168.61) million in 2011 [25], although more studies are needed to understand the total cost of dengue across Mexico.

### 3.1 Quality Assessment Tool

Overall study quality was assessed using a traffic light scoring system. Each criterion was scored as Yes = 1, No = −1, and N/A = 0, with total scores tabulated in Table 3. Items marked as N/A were excluded from the overall calculation to prevent skewing. Thresholds for overall assessment were ≥ 80% (green, good quality), ≥ 50 and < 80% (amber, fair quality), and < 50% (red, poor quality). The specific checklist applied is described in the Supporting Information S2.

**TABLE 3:** Quality assessment (*n* = 8).

First author	Year	Title	Quality
Undurraga [22]	2015	Economic and disease burden of dengue in Mexico	75.00
Zubieta‐Zavala [16]	2018	Economic impact of dengue in Mexico considering reported cases for 2012 to 2016	67.86
Shepard [26]	2013	Dengue in Mexico: Use of multiple data sources to estimate economic and disease burden.	46.43
Tiga. [20]	2016	Persistent symptoms of dengue: Estimates of the incremental disease and economic burden in Mexico.	52.00
Zubieta‐Zavala [23]	2016	Calculation of the average cost per case of dengue fever in Mexico using a micro‐costing approach	65.52
Legorreta‐Soberanis [24]	2017	Household costs of dengue illness: Secondary outcomes from a randomised controlled trial of dengue prevention in Guerrero state, Mexico	39.29
Shepard [21]	2011	Economic impact of dengue illness in the americas	46.43

### 3.2 Strengths and Limitations

This SLR presents a comprehensive review of published data on the economic burden of dengue in Mexico during 2010–2020. Costs were often stratified by admission type (outpatient and inpatient) and facility type (private, public, and first‐level to third‐level facilities), allowing for a clear overview of the differences in burden between these groups.

Dengue presents a broad spectrum of clinical manifestations, ranging from a self‐limiting febrile illness to severe disease with organ involvement and shock. The 1997 WHO classification categorised dengue as dengue fever (DF), dengue haemorrhagic fever (DHF), and dengue shock syndrome (DSS), whereas the 2009 revision introduced the categories of dengue without warning signs, dengue with warning signs, and severe dengue to improve the identification of severe cases and clinical management. Although signs and symptoms generally showed poor correlation with disease severity under both classification systems, hypotensive shock was strongly associated with severe disease and demonstrated near‐perfect agreement across both guidelines. Overall, the 1997 and 2009 classifications showed substantial concordance; however, the 2009 guidelines were found to be more sensitive for detecting severe dengue cases [27].

In addition to the above, a potential limitation of this study lies in how uncertainty was addressed in the reviewed literature. However, a structured and conservative approach was adopted: when a source of uncertainty could not be fully resolved, it was considered addressed if supported, even partially, by the best available evidence. While some residual uncertainty may persist—particularly when data were derived from different time periods or from the same institution—its impact was systematically minimised. Specifically, cost normalisation techniques were applied to adjust for temporal variation and economic fluctuations, thereby enhancing the comparability and robustness of the estimates.

Four studies (Shepard et al. [23, 25] Zubieta‐Zavala et al. [16] and Undurraga et al. [22]) investigated the economic burden by including expansion factors. Reported dengue cases were first adjusted using expansion factors. Costs were then estimated using the adjusted prevalence of dengue, which accounts for underreporting of cases and may therefore be more reflective of the true burden of dengue in Mexico. Undurraga et al. [22] was the only study that reported costs by age group. Adults appeared to have lost the most days due to illness (6.1 workdays), while children aged 5–14 and 15–18 years had the highest number of hospital visits (3.7 visits before hospitalisation). Therefore, the societal impact was distributed among all the age groups studied, although further research across different age groups is needed.

The main limitation of the SLR was the heterogeneous nature of the cost‐of‐illness studies. Significant differences were noted in data sources, methodology for calculating costs, and the definition of cost categories. In addition, several different settings (primary care, tertiary care, at‐home stay, specialist care) and diverse populations were studied, which complicates the comparison of the findings. The methodologies adopted to calculate productivity losses varied significantly between studies. This observation is compounded by the lack of consensus on how to value productivity loss in children monetarily [28]. Undurraga et al. [22] and Shepard et al. [23] adopted the human capital approach to estimate productivity loss, whereas the approach of Zubieta et al. [16] was highly subjective, with estimated indirect costs based on those reported by patients and caregivers themselves. Methodological disparities make it difficult to compare indirect cost estimates between studies. Zubieta et al. [16] reported productivity losses without quantifying the associated productivity costs.

Furthermore, the publications identified were limited in terms of the quality determined by the assessment tool of the National Health Service (NHS) Wales, as most publications (*n* = 4) did not perform a sensitivity analysis on key assumptions or point estimates, or they lacked sufficient detail on the evaluation of resource use and productivity losses (*n* = 3). Therefore, although this review provides a comprehensive overview of the published literature on the costs associated with dengue, further studies on the cost of illness are required to enable comparisons of the economic burden of dengue between settings and healthcare perspectives.

## 4 Discussion

The most important impact of dengue lies in the associated societal costs. In 2020, the COVID‐19 pandemic increased morbidity and delayed care for all diseases worldwide. The situation is similar for vector‐borne diseases, since promotion and prevention strategies were not developed in Mexico until recently.

Four cost‐of‐illness studies reported the societal impact of dengue from 2009 to 2017. Undurraga et al. [22] reported the number of hospital days for each level (primary to tertiary level), which amounted to 13.9 days in hospital, of which 7.4 days were in the acute phase and 6.5 days in the convalescent phase [22], potentially indicating a significant impact of dengue in Mexico, resulting in productivity loss. Although data were limited, all studies concluded that dengue has a significant impact on health expenditure and productivity losses.

Prevention of dengue is important because it reduces the incidence of a potentially severe and sometimes fatal disease, lessens the burden on healthcare systems, minimises the economic impact on individuals and communities, and helps avoid outbreaks that can strain public health resources and disrupt daily life, especially in endemic regions. Effective prevention strategies, including vaccination, mosquito control, and public awareness, would have a significant impact on the prevention and control of dengue [29]. Therefore, comprehensive management of the disease must take advantage of the recent tools, such as new dengue vaccines, to address the disease burden in Mexico.

Understanding the out‐of‐pocket cost of dengue is essential because it reveals the direct financial burden placed on individuals and families affected by the disease. These costs (which may include medical consultations, medications, transportation, and lost income) can be devastating for low‐income households. By quantifying these expenses, policymakers can design more equitable health interventions, improve financial protection, and strengthen health systems to reduce the economic vulnerability of at‐risk populations.

Several Latin American countries have reported detailed analyses of the economic burden of dengue during comparable periods. For example, Colombia recorded costs of US$167.8 million in 2010, US$129.9 million in 2011, and US$131.7 million in 2012 [30]. In Brazil, estimated annual costs ranged from US$164 million (90% CI: US$123–205 million) to US$447 million (90% CI: US$335–559 million) after adjustment for underreporting [31].

In this review, we found outbreak costs accounted for a substantial portion (30%–48%) of total economic burden. Notably, Zubieta‐Zavala et al. [23] reported that, in 2016, the cost of dengue prevention, surveillance, and control programs in Mexico was USD 11,766.52 per 10,000 inhabitants. Although frequently overlooked, these expenditures are vital for accurate assessment of government response and for understanding the true economic impact of dengue. To place these figures in a broader context, it is important to note that, in 2020, Mexico's gross domestic product (GDP) stood at USD 1.121 trillion, experiencing a 14.04% decrease from 2019. Correspondingly, health expenditure contracted from 2.6% of GDP in 2019 to 6.5% in 2020 [32]. These changes highlight the economic consequences of the COVID‐19 pandemic and underscores the need to consider these contextual factors in future analyses of public health costs and resource allocation. The persistence of such economic pressures may influence the capacity to implement dengue control measures in the coming years.

Finally, the fight against dengue in Mexico has been intensified through comprehensive public policies aim to significantly reduce the incidence of the disease. The National Plan for the Control of Dengue and Other Arboviruses 2025–2030 is the government's main strategy in this area [33]. The primary objective of the programme is to reduce cases of dengue, zika, and chikungunya in the country by 50% by 2030. To achieve this, coordinated actions have been established at the federal, state, and municipal levels, focussing on prevention, vector control, and community engagement. Despite the efforts, dengue remains a public health challenge in Mexico. Disease control is complicated by factors such as climate change, uncontrolled urbanisation, and insecticide resistance in mosquitoes. However, the implementation of sound public policies and collaboration between government, local communities, and the scientific community offer a promising avenue for reducing the burden of dengue in Mexico.

## 5 Conclusions

Given that different data sources and variables were used, not all the studies in this SLR can be directly compared to estimate the economic burden of dengue in Mexico. Nonetheless, we have thoroughly examined the current literature to bridge the gap in our understanding of the economic impact of dengue in Mexico during the study period.

Recent events, such as the COVID‐19 pandemic, have shown that underreporting of the disease during a major health emergency can create a misleading impression that the incidence of dengue and other transmissible infections is low (as was the case). However, in countries such as Mexico, where dengue is endemic and often co‐occurs with COVID‐19, mortality rates could rise, and the healthcare system could face additional challenges. Integrated programs that incorporate both economic and epidemiological information are essential, potentially merging health institutions (IMSS, ISSSTE, and MoH) to facilitate a more cohesive response, given that dengue is the most significant vector‐borne disease and the one with the greatest economic impact in Mexico.

## Funding

The systematic literature review was funded by Takeda Pharmaceuticals.

## Ethics Statement

The authors have nothing to report.

## Conflicts of Interest

Gilberto Sánchez‐González and Luz del C. Hernández‐Hernández have no conflicts of interest to declare. Elaine Gallagher was an employee of Takeda at the time this research was performed.

## Supporting information


Supporting Information S1

Supporting Information S2

## Data Availability

The data that supports the findings of this study are available in the supplementary material of this article.
