# Supplementary material for: Economic Impact of Dengue Virus in Mexico Over One Decade: A Systematic Literature Review (2010–2020)
Source: Int J Health Plann Manage. 2026 Jul 28;41(5):1085–93. doi: 10.1002/hpm.70096 (PMC13551542; doi:10.1002/hpm.70096)
Supplement: Supplementary file 1 [file HPM-41-1085-s001.docx]

## Quality assessment checklist

The NHS Wales checklist to assist with the critical appraisal of cost of illness studies will be used to assess the quality of studies that focus on cost data. The questions included in the checklist are outlined below:

1. What was the motivation of the study?
2. What was the perspective of the study?
3. Was the appropriate epidemiologic approach taken?
4. Was the study question well specified?
5. Were all relevant, non-trivial cost components and their stakeholders identified?
6. Were necessary timeframes specified?
7. Was a case of disease or risk factor adequately and appropriately defined?
8. Was the counterfactual population occurrence plausible and meaningful?
9. Was an appropriate method(s) of quantification used, such that additional, or excess, costs were measured?
10. Was an appropriate method(s) of quantification used, such that only costs specific to (caused by) the health problem were included (confounders controlled)?
11. Was an appropriate method(s) of quantification used, such that all important effects were captured?
12. Was an appropriate method(s) of quantification used, such that important differences across subpopulations were accounted for?
13. Was an appropriate method(s) of quantification used, such that the required level of detail could be provided?
14. Was the resource quantification method(s) well executed?
15. For population-based studies, were cost allocation methods, data and assumptions valid?
16. For person-based studies, were appropriate statistical tests performed and reported?
17. Were data representative of the study population?
18. Were there any other relevant resource quantification issues?
19. Were healthcare resources valued appropriately?
20. Was the approach for valuing production losses justified, and assumptions valid?
21. Was the inclusion of intangible costs appropriate?
22. Was double counting of mortality-related production losses avoided?
23. Were losses valued appropriately, given the study’s perspective?
24. Did the analysis address the study question?
25. Was a range of estimates presented?
26. Were the main uncertainties identified?
27. Was a sensitivity analysis performed on important (uncertain) parameter estimates?
28. Was a sensitivity analysis performed on key assumptions? (including the counterfactual)
29. Was a sensitivity analysis performed on point estimates? (based on confidence or credible intervals)
30. Was adequate documentation and justification given for cost components, data and sources, assumptions and methods?
31. Was uncertainty around the estimates and its implications adequately discussed?
32. Were important limitations discussed regarding the cost components, data, assumptions and methods?
33. Were the results presented at the appropriate level of detail to answer the study question (cost components; disease subtypes, severity, stage; subpopulation groups, cost bearers)?

List of tables

Table 1. Embase search strategy: cost data.

| Search line | Search terms | Hits 17/03/2020 |
| --- | --- | --- |
| **Disease** | | |
| **1** | Dengue/ or severe dengue/ or dengue virus/ or dengue haemorrhagic fever/ or dengue shock syndrome/ or dengue virus 1/ or dengue virus 2/ or dengue virus 3/ or dengue virus 4/ | 26,304 |
| **2** | (Dengue or severe dengue or dengue virus or dengue haemorrhagic fever or alarm signs).mp | 30,271 |
|  | | |
| **3** | Health care cost/ or cost of illness/ or hospitalization/ or hospital costs/ or hospital cost/ or economic aspect/ or prevention cost or prevention costs or prevention or vector control or outbreak control or outbreak prevention | 1,850,350 |
| **4** | (Economic aspect or financial aspect or cost of care or direct cost* or drug cost* or medical cost* or physician cost* or nurse cost* or clinic visit* or hospital visit* or cost* or average wage).mp | 1,061,871 |
| **5** | Caregiver burden/ or caregiver/ or caregivers/ or medical leave/ or sick leave/ or health care financing/ or healthcare financing/ or health expenditures/ or drug cost/ or medical fee/ or fees, medical/ or fees, pharmaceutical/ or hospital charge/ or work disability/ or absenteeism/ or productivity/ or medical leave/ or catastrophic financial shock or financial shock or household cost* or family cost* or catastrophic financial cost* or financial cost* | 392,868 |
| **6** | (Economic burden or indirect cost* or productivity or societal burden or societal cost* or resource utilization or resource u* or utilit* or workdays lost or school days lost).mp | 406,803 |
|  | | |
| **7** | 1 or 2 | 30,271 |
| **8** | 3 or 4 or 5 or 6 | 2,968,177 |
| **9** | 7 and 8 | 7,720 |
| **10** | 9 and (Mexico).mp | 255 |
| **11** | **TOTAL** | **153** |

Table 2. MEDLINE search strategy: cost data.

| Search line | Search terms | Hits 17/03/2020 |
| --- | --- | --- |
| **Disease** | | |
| **1** | Dengue/ or severe dengue/ or dengue virus/ or dengue hemorrhagic fever/ or dengue shock syndrome/ or dengue virus 1/ or dengue virus 2/ or dengue virus 3/ or dengue virus 4/ | 15,345 |
| **2** | (Dengue or severe dengue or dengue virus or dengue hemorrhagic fever or alarm signs).mp | 22,379 |
| **Cost of illness/economic burden** | | |
| **3** | Health care cost/ or cost of illness/ or hospitalization/ or hospital costs/ or hospital cost/ or economic aspect/ or prevention cost or prevention costs or prevention or vector control or outbreak control or outbreak prevention | 1,667,767 |
| **4** | (Economic aspect or financial aspect or cost of care or direct cost* or drug cost* or medical cost* or physician cost* or nurse cost* or clinic visit* or hospital visit* or cost* or average wage).mp | 686,093 |
| **5** | caregiver burden/ or caregiver/ or caregivers/ or medical leave/ or sick leave/ or health care financing/ or healthcare financing/ or health expenditures/ or drug cost/ or medical fee/ or fees, medical/ or fees, pharmaceutical/ or hospital charge/ or work disability/ or absenteeism/ or productivity/ or medical leave/ or catastrophic financial shock or financial shock or household cost* or family cost* or catastrophic financial cost* or financial cost* | 108,014 |
| **6** | (Economic burden or indirect cost* or productivity or societal burden or societal cost* or resource utilization or resource u* or utility* or workdays lost or school days lost).mp | 284,921 |
|  | | |
| **7** | 1 or 2 | 22,379 |
| **8** | 3 or 4 or 5 or 6 | 2,496,405 |
| **9** | 7 and 8 | 5,171 |
| **10** | 9 and (Mexico).mp | 191 |
| **11** | **TOTAL** | **102** |

Table 3. Evidence-Based Medicine review databases search strategy: cost data.

| Search line | Search terms | Hits 17/03/2020 |
| --- | --- | --- |
| **Disease** | | |
| **1** | Dengue/ or severe dengue/ or dengue virus/ or dengue hemorrhagic fever/ or dengue shock syndrome/ or dengue virus 1/ or dengue virus 2/ or dengue virus 3/ or dengue virus 4/ | 298 |
| **2** | (Dengue or severe dengue or dengue virus or dengue hemorrhagic fever or alarm signs).mp | 819 |
|  | | |
| **3** | Health care cost/ or cost of illness/ or hospitalization/ or hospital costs/ or hospital cost/ or economic aspect/ or prevention cost or prevention costs or prevention or vector control or outbreak control or outbreak prevention | 122,561 |
| **4** | (Economic aspect or financial aspect or cost of care or direct cost* or drug cost* or medical cost* or physician cost* or nurse cost* or clinic visit* or hospital visit* or cost* or average wage).mp | 113,523 |
| **5** | Caregiver burden/ or caregiver/ or caregivers/ or medical leave/ or sick leave/ or health care financing/ or healthcare financing/ or health expenditures/ or drug cost/ or medical fee/ or fees, medical/ or fees, pharmaceutical/ or hospital charge/ or work disability/ or absenteeism/ or productivity/ or medical leave/ or catastrophic financial shock or financial shock or household cost* or family cost* or catastrophic financial cost* or financial cost* | 6,265 |
| **6** | (Economic burden or indirect cost* or productivity or societal burden or societal cost* or resource utilization or resource u* or utility* or workdays lost or school days lost).mp | 35,083 |
|  | | |
| **7** | 1 or 2 | 819 |
| **8** | 3 or 4 or 5 or 6 | 234,776 |
| **9** | 7 and 8 | 267 |
| **10** | 9 and (Mexico).mp | 40 |
| **11** | **TOTAL** | **37** |

Table 4. SciELO search strategy: cost data.

| Search line | Search terms | Hits 13/03/2020 |
| --- | --- | --- |
| **Disease** | | |
| **1** | Dengue | 2,306 |
| **2** | Dengue and Mexico | 114 |
| **3** | **TOTAL** | 77 |

Table 5. LILACS search strategy: cost data.

| Search line | Search terms | Hits 13/03/2020 |
| --- | --- | --- |
| **Disease** | | |
| **1** | Dengue | 3,463 |
| **2** | Dengue and Mexico | 114 |
| **3** | **TOTAL** | 45 |

Table 6. Cost normalization inflation rates.

| **Year of costs** | **Inflation rates** |
| --- | --- |
| 2010 | 1.1724373555417 |
| 2011 | 1.13656146777571 |
| 2012 | 1.11351777485474 |
| 2013 | 1.09744287572385 |
| 2014 | 1.07992447283049 |
| 2015 | 1.07864414788813 |
| 2016 | 1.06520643147908 |
| 2017 | 1.04298710835509 |
| 2018 | 1.01811976567758 |

Source: US Inflation Calculator. <https://www.usinflationcalculator.com/inflation/consumer-price-index-and-annual-percent-changes-from-1913-to-2008/>^[1]^

**Table 7**. Estimated average economic burden of dengue illness per episode (ambulatory cases).

| **Subgroup** | **Direct medical cost**  **(95% CI or ±SD)** | **Direct non-medical cost**  **(95% CI or ±SD)** | **Indirect cost**  **(95% CI or ±SD)** | **Outbreak cost**  **(95% CI or ±SD)** | **Total cost**  **(95% CI, or ±SD)** |
| --- | --- | --- | --- | --- | --- |
| **Shepard et al 2013, 2012 costing year, societal perspective** | | | | | |
| 2010 | NR | NR | NR | NR | $123.46 million |
| 2011 | NR | NR | NR | NR | $45.61 million |
| **Shepard et al 2011, 2010 costing year, societal perspective** | | | | | |
| NR | $309.52 estimated average cost per dengue case | $15.24 estimated average cost per dengue case | $245.04 estimated average cost per dengue case | NR | $569.80 estimated average cost per dengue case |
| **Zubieta-Zavala et al 2016, 2014 costing year, 1,168 patients, healthcare system perspective** | | | | | |
| Ideal costs: Secretariat of Health (US$) | $178.96 average cost per case | NR | NR | NR | NR |
| Real costs: Secretariat of Health (US$) | $35.20 average cost per case | NR | NR | NR | NR |
| Ideal costs: Mexican Social Security Institute (US$) | $364.47 average cost per case | NR | NR | NR | NR |
| Real costs: Mexican Social Security Institute (US$) | $99.38 average cost per case | NR | NR | NR | NR |
| **Zubieta-Zavala et al 2016, 2014 costing year, 1,168 patients/53% females, patient perspective** | | | | | |
| Secretariat of Health (US$) | $17.59 average cost per case ($10.45, $28.51) | NR | NR | NR | NR |
| Mexican Social Security Institute (US$) | $42.51 average cost per case ($18.68, $67.13) | NR | NR | NR | NR |
| **Tiga et al 2016, 2012 costing year, societal perspective** | | | | | |
| Acute dengue illness | $45.92 million per year | NR | $14.15 million per year | NR | $60.07 million per year |
| **Undurraga et al 2015, 2012 costing year, societal perspective** | | | | | |
| Homecare or pharmacy | $19.18 per ambulatory visit | NR | NR | NR | NR |
| Health center (no beds) | $75.57 per ambulatory visit | NR | NR | NR | NR |
| Primary-level hospital | $93.33 per ambulatory visit | NR | NR | NR | NR |
| Secondary-level hospital | $106.34 per ambulatory visit | NR | NR | NR | NR |
| Tertiary-level hospital | $110.82 per ambulatory visit | NR | NR | NR | NR |
| Best estimate, unit cost per ambulatory visit | $72.97 per ambulatory visit | $10.12 per bed day (average) | $12.17 per day 2010, | NR | NR |
|  |  |  | $12.31 per day 2011 |  |  |
| Best estimate, unit cost per ambulatory visit | $72.97 best estimate per ambulatory visit | $10.12 per bed day (average) | $8.15 per day 2010, | NR | NR |
|  |  |  | $8.45 per day 2011 |  |  |
| Best estimate, unit cost per ambulatory visit | $72.97 per ambulatory visit | $10.12 per bed day (average) | $10.07 per day 2010, | NR | NR |
|  |  |  | $10.17 per day 2011 |  |  |
| Non-fatal dengue episode | $281.72 per episode | $102.44 per episode | $118.03 per episode | NR | $502 per episode |
| NR | NR | NR | NR | NR | $87.89 million annual cost 2010 ($55.86, $252.62 million) |
| NR | NR | NR | NR | NR | $32.25 million annual cost 2011 ($20.53, $92.76 million) |
| Non-fatal dengue episode (costing year 2011) | $38.53 million annual cost | $9.91 million annual cost | $10.69 million annual cost | NR | $165.8807429 million annual cost 2011 (includes indirect fatal costs, and surveillance ‡ vector control costs) ($151.26, $256.78 million) |
| NR | NR | NR | NR | NR | $60.07 million average annual cost 2010–2011 ($38.17, $172.76 million) |
| **Zubieta-Zavala et al 2018, 2012 costing year, 84,612 patients, healthcare systemand patient** **perspectives** | | | | | |
| NR | $6,752,417 average cost† | NR | NR | NR | $115,546 average cost of dengue ($50,773, $182,432) ‡ |
| **Zubieta-Zavala et al 2018, 2013 costing year, 125,795 patients, healthcare system**† **and patient**‡ **perspectives** | | | | | |
| NR | $11,354,840 average cost† | NR | NR | NR | $194,302 Average cost of dengue ($85,380, $306,777) ‡ |
| **Zubieta-Zavala et al 2018, 2014 costing year, 54,948 patients, healthcare system and patient** **perspectives** | | | | | |
| NR | $5,005,644 average cost† | NR | NR | NR | $85,655 Average cost of dengue ($37,638, $135,238) ‡ |
| **Zubieta-Zavala et al 2018, 2015 costing year, 67,336 patients, healthcare system and patient perspectives** | | | | | |
| NR | $6,894,647 annual cost† | NR | NR | NR | $117,979 Average cost of dengue ($51,842, $186,274) ‡ |
| **Zubieta-Zavala et al 2018, 2016 costing year, 45,624 patients, healthcare system and patient perspectives** | | | | | |
| NR | $4,762,510 annual cost† | NR | NR | NR | $81,494 Average cost of dengue ($35,811, $128,670) ‡ |

CI: confidence interval; NR: not reported; SD: standard deviation; US: United States; †: heathcare system; ‡: patient.

**Table 8**. Per-episode economic burden of dengue illness, including hospitalized and ICU cases, as reported in the selected literature.

| **Subgroup** | **Cost type** | **Direct medical cost**  **(95% CI or ±SD)** | **Direct non-medical cost**  **(95% CI or ±SD)** | **Indirect cost**  **(95% CI or ±SD)** | **Outbreak cost**  **(95% CI or ±SD)** | **Total cost**  **(95% CI, or ±SD)** |
| --- | --- | --- | --- | --- | --- | --- |
| **Shepard et al 2013, 2012 costing year, societal perspective** | | | | | | |
| 2010 | Hospitalization | NR | NR | NR | NR | $38.61 million |
| 2011 | Hospitalization | NR | NR | NR | NR | $36.64 million |
| **Shepard et al 2011, 2010 costing year, societal perspective** | | | | | | |
| NR | Hospitalization | $588.56 Estimated average cost per dengue case | $168.83 Estimated average cost per dengue case | $660.08 Estimated average cost per dengue case | NR | $1,417.47 Estimated average cost per dengue case |
| **Zubieta-Zavala et al 2016, 2014 costing year, 1,168 patients, healthcare system perspective** | | | | | | |
| Ideal costs: Secretariat of Health (US$) | Hospitalization | $634.74 average cost per case | NR | NR | NR | NR |
| Real costs: Secretariat of Health (US$) | Hospitalization | $530.16 average cost per case | NR | $178.66 per case (patient and caregiver combined) | NR | NR |
| Ideal costs: Mexican Social Security Institute (US$) | Hospitalization | $2,205.79 average cost per case | NR | NR | NR | NR |
| Real costs: Mexican Social Security Institute (US$) | Hospitalization | $1,776.14 average cost per case | NR | $574.97 per case (patient and caregiver combined) | NR | NR |
| Ideal costs: Secretariat of Health (US$) | ICU | $7,328.57 average cost per case | NR | NR | NR | NR |
| Real costs: Secretariat of Health (US$) | ICU | $5,790.04 average cost per case | NR | $165.75 per case (patient and caregiver combined) | NR | NR |
| Ideal costs: Mexican Social Security Institute (US$) | ICU | $25,327.07 average cost per case | NR | NR | NR | NR |
| Real costs: Mexican Social Security Institute (US$) | ICU | $10,123.79 average cost per case | NR | $314.59 per case (patient and caregiver combined) | NR | NR |
| **Zubieta-Zavala et al 2016, 2014 costing year, 1,168 patients, 53% females, patient perspective** | | | | | | |
| Secretariat of Health (US$) | Hospitalization | $103.58 average cost per case ($73.01, $141.98) | $82.05 per case ($64.64, $103.13) | Patient: $102.97 ($73, $144.61) Caregiver: $78.21 ($47.43, $131.5) per case | NR | NR |
| Mexican Social Security Institute (US$) | Hospitalization | $103.29 average cost per case ($52.11, $177.79) | $93.05 per case ($ 68.25, $142.87) | Patient: $132.62 ($103.72, $177.72) Caregiver: $406.06 ($132.14, $872.15) per case | NR | NR |
| **Tiga et al 2016, 2012 costing year, societal perspective** | | | | | | |
| Acute dengue illness | Hospitalization | $25.12 million per year | NR | $3.02 million per year | NR | $28.14 million per year |
| **Undurraga et al 2015, 2012 costing year, societal perspective** | | | | | | |
| Primary-level hospital | Hospitalization | $256.7 per bed day | NR | NR | NR | NR |
| Secondary-level hospital | Hospitalization | $267.82 per bed day | NR | NR | NR | NR |
| Tertiary-level hospital | Hospitalization | $346.31 per bed day | NR | NR | NR | NR |
| Best estimate, unit cost per bed day | Hospitalization | $267.29 per bed day | NR | $12.17 per day 2010, | NR | NR |
|  |  |  |  | $12.31 per day 2011 |  |  |
| Best estimate, unit cost per bed day | Hospitalization | $267.29 best estimate per bed day | NR | $8.15 per day 2010, | NR | NR |
|  |  |  |  | $8.45 per day 2011 |  |  |
| Best estimate, unit cost per bed day | Hospitalization | $267.29 per bed day | NR | $10.07 per day 2010, | NR | NR |
|  |  |  |  | $10.17 per day 2011 |  |  |
| Non-fatal dengue episode | Hospitalization | $1124.65 per episode | $193.7 per episode | $159.23 per episode | NR | $1,477 per episode |
| NR | Hospitalization | NR | NR | NR | NR | $29.38 million annual cost 2010 ($15.9, $91.76 million) |
| NR | Hospitalization | NR | NR | NR | NR | $26.89 million annual cost 2011 ($14.49, $84.17 million) |
| Non-fatal dengue episode | Hospitalization | $71.71 million annual cost (2010) | $21.93 million annual cost (2010) | $23.61 million annual cost (2010) | NR | $212.0694602 million annual cost 2010 (includes indirect fatal costs, and surveillance ‡ vector control costs) ($183.18, $397.48 million) |
| NR | Hospitalization | NR | NR | NR | NR | $28.14 million average 2010–2011 ($15.18, $87.94 million) |
| **Zubieta-Zavala et al 2018, 2012 costing year, 84,612 patients, healthcare system and patient perspectives** | | | | | | |
| NR | Hospitalization | $32,569,467 average cost† | NR | NR | NR | $5,121,860 average cost of dengue ($2,482,217, $9,550,134) ‡ |
| NR | ICU | $9,770,795 average cost† | NR | NR | NR | $254,882 Average cost of dengue ($164,858, $415,597) ‡ |
| **Zubieta-Zavala et al 2018, 2013 costing year, 125,795 patients, healthcare system and patient perspectives** | | | | | | |
| NR | Hospitalization | $36,058,818 average cost† | NR | NR | NR | $5,670,593 Average cost of dengue ($2,748,151, $10,573,293) ‡ |
| NR | ICU | $10,817,596 average cost† | NR | NR | NR | $282,188 Average cost of dengue ($182,520, $460,122) ‡ |
| **Zubieta-Zavala et al 2018, 2014 costing year, 54,948 patients, healthcare system and patient perspectives** | | | | | | |
| NR | Hospitalization | $16,328,651 average cost† | NR | NR | NR | $2,567,836 Average cost of dengue ($1,244,456, $4,787,944) ‡ |
| NR | ICU | $4,898,573 average cost† | NR | NR | NR | $127,784 Average cost of dengue ($82,650, $208,359) ‡ |
| **Zubieta-Zavala et al 2018, 2015 costing year, 67,336 patients, healthcare system and patient perspectives** | | | | | | |
| NR | Hospitalization | $10,671,721 annual cost† | NR | NR | NR | $1,678,230 Average cost of dengue ($813,323, $3,129,199) ‡ |
| NR | ICU | $3,201,502 annual cost† | NR | NR | NR | $83,515 Average cost of dengue ($54,017, $136,174) ‡ |
| **Zubieta-Zavala et al 2018, 2016 costing year, 45,624 patients, healthcare system and patient perspectives** | | | | | | |
| NR | Hospitalization | $40,878,315 annual cost† | NR | NR | NR | $1,127,812 Average cost of dengue ($546,574, $2,102,901) ‡ |
| NR | ICU | $2,151,490 annual cost† | NR | NR | NR | $56,123 Average cost of dengue ($36,301, $91,512) ‡ |

CI: confidence interval; ICU: intensive care unit; NR: not reportedSD: standard deviation; US: United States.

**Table 9**. Estimated average economic burden of dengue illness per episode (direct and indirect costs, fatal cases, and other components of the surveillance programs).

| **Subgroup** | **Cost type** | **Direct medical cost**  **(95% CI or ±SD)** | **Direct non-medical cost**  **(95% CI or ±SD)** | **Indirect cost**  **(95% CI or ±SD)** | **Outbreak cost**  **(95% CI or ±SD)** | **Total cost**  **(95% CI, or ±SD)** |
| --- | --- | --- | --- | --- | --- | --- |
| **Shepard et al 2013, 2012 costing year, societal perspective** | | | | | | |
| 2010 | Fatal | NR | NR | NR | NR | $26.55 million |
| 2011 | Fatal | NR | NR | NR | NR | $25.67 million |
| 2010 | Surveillance & vector control | NR | NR | NR | NR | $86.07 million |
| 2011 | Surveillance & vector control | NR | NR | NR | NR | $98.59 million |
| 2010 | Annual economic cost | $89.64 million | $21.94 million | $50.44 million non-fatal dengue | $86.07 million cost for surveillance ‡ vector control | $274.7 million aggregate economic cost |
| 2011 | Annual economic cost | $45.77 million | $9.91 million | $26.5 non-fata dengue | $98.55 million cost for surveillance ‡ vector control | $206.56 million aggregate economic cost ($188.18, $321.81 million) |
|  |  |  |  | $25.61 fatal dengue |  |  |
| **Zubieta-Zavala et al 2016, 2014 costing year, 1,168 patients, healthcare system perspective** | | | | | | |
| Ideal costs: Secretariat of Health (US$) | Ambulatory, hospitalization, and ICU | $8,142.28 average cost per case | NR | NR | NR | NR |
| Real costs: Secretariat of Health (US$) | Ambulatory, hospitalization, and ICU | $6,355.42 average cost per case | NR | NR | NR | NR |
| Ideal costs: Mexican Social Security Institute (US$) | Ambulatory, hospitalization, and ICU | $27,897.33 average cost per case | NR | NR | NR | NR |
| Real costs: Mexican Social Security Institute (US$) | Ambulatory, hospitalization, and ICU | $11,999.32 average cost per case | NR | NR | NR | NR |
| **Tiga et al 2016, 2012 costing year, societal perspective** | | | | | | |
| Persistent symptoms | NR | $2.17 million per year ($0.7–$3.2 million) | NR | $23.03 million per year ($12.49–$30.65 million) | NR | $25.21 million per year ($14.48–$32.80 million) |
| Fatal acute dengue illness | NR | NR | NR | $8.43 million per year | NR | NR |
| Total acute dengue illness | NR | $71.04 million per year ($29.23–$131.15 million) | NR | $25.60 million per year ($12.48–$46.11 million) | NR | $96.64 million per year ($74.97–$232.26 million) |
| **Undurraga et al 2015, 2012 costing year, societal perspective** | | | | | | |
| Fatal dengue | Ambulatory and hospitalization | NR | NR | NR | NR | $8.71 million annual cost 2010 ($5.46, $12.50 million) |
| Fatal dengue | Ambulatory and hospitalization | NR | NR | NR | NR | $8.15 million annual cost 2011 ($5.12, $11.70 million) |
| Fatal dengue | Ambulatory and hospitalization | NR | NR | NR | NR | $8.43 million average 2010–2011 ($5.29, $12.10 million) |
| **Zubieta-Zavala et al 2018, 2012 costing year, 84,612 patients, healthcare system**† **and patient**‡ **perspectives** | | | | | | |
| NR | Ambulatory, hospitalized, and ICU | $49,092,680 average use cost† | NR | NR | NR | $5,492,287 Average cost of dengue ($2,697,850, $10,148,163) ‡ |
| Health facility | Ambulatory, hospitalization, and ICU | $84,260,310 average use cost† | NR | NR | NR | $7,954,959 Average cost of dengue ($3,893,767, $14,627,022) ‡ |
| All symptoms | Ambulatory, hospitalization, and ICU | $122,494,061 average use cost† | NR | NR | NR | $11,400,541 Average cost of dengue ($5,578,482, $20,953,081) ‡ |
| **Expansion factor analysis for 2012** | | | | | | |
| Without expansion factor | Ambulatory, hospitalization, and ICU | NR | NR | NR | NR | $160,861,018 |
| Health facility Expansion factors: 3.7 ambulatory and 1.4 hospitalization |  |  |  |  |  | $198,491,321 |
| All symptoms Expansion factors: 5.6 ambulatory and 2.0 hospitalization |  |  |  |  |  | $240,170,653 |
| **Zubieta-Zavala et al 2018, 2013 costing year, 125,795 patients, healthcare system and patient perspectives** | | | | | | |
| NR | Ambulatory, hospitalization, and ICU | $58,231,255 average use cost† | NR | NR | NR | $6,147,083 Average cost of dengue ($3,016,052, $11,340,193) ‡ |
| Health facility | Ambulatory, hospitalization, and ICU | $107,639,890 average use cost† | NR | NR | NR | $9,052,811 Average cost of dengue ($4,418,848, $16,581,858) ‡ |
| All symptoms | Ambulatory, hospitalization, and ICU | $157,339,935 average use cost† | NR | NR | NR | $12,993,655 Average cost of dengue ($6,339,474, $23,784,783) ‡ |
| **Expansion factor analysis for 2013** | | | | | | |
| Without expansion factor | Ambulatory, hospitalization, and ICU | NR | NR | NR | NR | $176,816,737 |
| Health facility Expansion factors: 3.7 ambulatory and 1.4 hospitalization |  |  |  |  |  | $229,131,101 |
| All symptoms Expansion factors: 5.6 ambulatory and 2.0 hospitalization |  |  |  |  |  | $282,771,988 |
| **Zubieta-Zavala et al 2018, 2014 costing year, 54,948 patients, healthcare system and patient perspectives** | | | | | | |
| NR | Ambulatory, hospitalization, and ICU | $26,232,869 average use cost† | NR | NR | NR | $2,781,276 Average cost of dengue ($1,364,745, $5,131,542) ‡ |
| Health facility | Ambulatory, hospitalization, and ICU | $48,239,000 average use cost† | NR | NR | NR | $4,090,794 Average cost of dengue ($1,997,214, $7,495,209) ‡ |
| All symptoms | Ambulatory, hospitalization, and ICU | $70,486,060 average use cost† | NR | NR | NR | $5,870,913 Average cost of dengue ($2,864,992, $10,749,945) ‡ |
| **Expansion factor analysis for 2014** | | | | | | |
| Without expansion factor | Ambulatory, hospitalization, and ICU | NR | NR | NR | NR | $144,288,132 |
| Health facility Expansion factors: 3.7 ambulatory and 1.4 hospitalization |  |  |  |  |  | $167,603,781 |
| All symptoms Expansion factors: 5.6 ambulatory and 2.0 hospitalization |  |  |  |  |  | $191,630,959 |
| **Zubieta-Zavala et al 2018, 2015 costing year, 67336 patients, healthcare system and patient perspectives** | | | | | | |
| NR | Ambulatory, hospitalization, and ICU | $20,767,870 average use cost† | NR | NR | NR | $1,879,725 Average cost of dengue ($919,184, $3,451,649) ‡ |
| Health facility | Ambulatory, hospitalization, and ICU | $44,932,707 average use cost† | NR | NR | NR | $2,902,967 Average cost of dengue ($1,406,096, $5,260,740) ‡ |
| All symptoms | Ambulatory, hospitalization, and ICU | $59,953,467 average use cost† | NR | NR | NR | $4,184,177 Average cost of dengue ($2,025,003, $7,573,886) ‡ |
| **Expansion factor analysis for 2015** | | | | | | |
| Without expansion factor | Ambulatory, hospitalization, and ICU | NR | NR | NR | NR | $141,765,309 |
| Health facility Expansion factors: 3.7 ambulatory and 1.4 hospitalization |  |  |  |  |  | $166,953,390 |
| All symptoms Expansion factors: 5.6 ambulatory and 2.0 hospitalization |  |  |  |  |  | $183,088,329 total economic impact |
| **Zubieta-Zavala et al 2018, 2016 costing year, 45,624 patients, healthcare system and patient perspectives** | | | | | | |
| NR | Ambulatory, hospitalization, and ICU | $47,792,315 or $14,085,667 Average use cost (two different values in different sections of the publication) † | NR | NR | NR | $1,265,432 Average cost of dengue ($618,685, $2,323,084) ‡ |
| Health facility | Ambulatory, hospitalization, and ICU | $26,459,409 Average use cost† | NR | NR | NR | $1,959,043 Average cost of dengue ($948,525, $3,548,259) ‡ |
| All symptoms | Ambulatory, hospitalization, and ICU | $41,013,389 Average use cost† | NR | NR | NR | $2,824,246 Average cost of dengue ($1,366,291, $5,109,380) ‡ |
| **Expansion factor analysis for 2016** | | | | | | |
| Without expansion factor | Ambulatory, hospitalization, and ICU | NR | NR | NR | NR | $138,696,003 |
| Health facility Expansion factors: 3.7 ambulatory and 1.4 hospitalization |  |  |  |  |  | $155,977,654 |
| All symptoms Expansion factors: 5.6 ambulatory and 2.0 hospitalization |  |  |  |  |  | $167,070,292 |

CI: confidence interval; ICU: intensive care unit; NR: not reported, SD: standard deviation; US: United States.
